# Emerging therapies for PBC

**DOI:** 10.1007/s00535-020-01664-0

**Published:** 2020-01-22

**Authors:** David Maxwell Hunter Chascsa, Keith Douglas Lindor

**Affiliations:** 1 Department of Gastroenterology and Hepatology, Mayo Clinic, MD 5777 E. Mayo Blvd, Phoenix, AZ 85054 USA; 2 Department of Transplant Center, Mayo Clinic, MD 5777 E. Mayo Blvd, Phoenix, AZ 85054 USA; 3 Office of University Provost, Arizona State University, MD, 550 North 3rd Street, Phoenix, AZ 85004 USA

**Keywords:** Primary biliary cholangitis, Ursodeoxycholic acid, Obeticholic acid, Fibrate, Liver disease

## Abstract

Primary biliary cholangitis is an uncommon cholestatic liver disease predominantly affecting middle-aged women. Left untreated, there is a high risk of progression to end-stage liver disease. Few treatment options exist. To date, ursodeoxycholic acid (UDCA) and obeticholic acid (OCA) are the only medical therapies approved for use, other than symptomatic treatments and liver transplantation, the latter of which is reserved for those developing complications of cirrhosis or with intractable pruritus. UDCA improves outcomes, but many patients do not adequately respond. OCA therapy may improve response, but long-term data are limited. New therapies are desperately needed, but evaluation has been limited by the fact that the disease is heterogeneous, hard end points take years to develop, and there are different criteria in use for determining therapeutic response based on surrogate biomarkers. Fibrates appear to be the most promising new therapy and have beneficially affected surrogate end points and are beginning to show improvement in clinical end points.

## Background

Primary biliary cholangitis (PBC) is an uncommon cholestatic liver disease predominantly affecting women with an overall prevalence of 6–402 per million persons; incidence may be on the rise [1–5]. The age of onset is typically in the fourth or fifth decade of life [6–8]. The exact pathophysiologic basis for the development of PBC is yet to be fully elucidated; however, evidence supports an autoimmune-induced mechanism. This is supported by the presence of a hallmark autoantibody: anti-mitochondrial antibody (AMA), in nearly all PBC patients. Genetic risk and environmental exposures have also been implicated [6–14].

## Diagnosis of PBC

Diagnosis of PBC relies on fulfillment of two of three hallmark criteria [15–18]. These include a persistently elevated alkaline phosphatase (ALP) in the absence of other hepatobiliary pathology, detection of a positive anti-mitochondrial antibody (AMA), and/or a liver biopsy with characteristic findings [17].

After identification of an elevated ALP, assessment should be made to detect the presence or absence of an elevated AMA. AMA is the hallmark antibody of PBC, and is found in over 95% of patients with the disease [12, 19–27]. Approximately, 5% of PBC patients will lack a detectable AMA. Biopsy is not necessary for diagnosis and may be reserved for patients with high clinical suspicion but negative AMA, or to exclude coexistent liver disease such as autoimmune hepatitis.

**Fig. 1:**
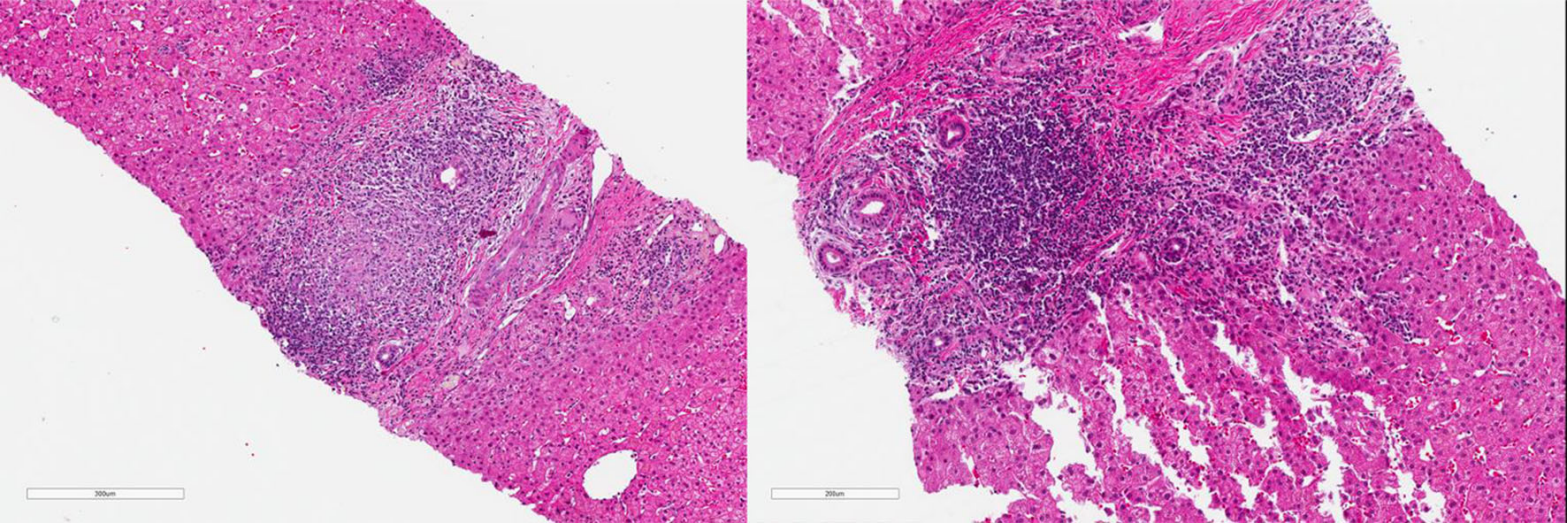
Histologic features of PBC with overlap. The image demonstrates a classic “florid duct lesion” of PBC. Typically, a biopsy is not required to make the diagnosis of PBC. In this particular case, a 70-year-old woman presented with a long-standing alkaline phosphatase elevation 2–3 times the upper limit of normal. AMA testing was negative, but ANA was markedly positive at 4.6 units. Abdominal ultrasound was pursued showing findings suspicious for cirrhosis, but no bile duct anomaly. Diagnostic liver biopsy was pursued. Interestingly, the case demonstrates an overlap syndrome as well, given the presence of interface hepatitis. This highlights the need for diagnostic liver biopsy in cases of AMA-negative but suspected PBC, and when there is concern for an overlap syndrome. It further highlights the utility of ANA assessment in patients suspected of PBC

Histologically, PBC is characterized by non-suppurative inflammation of the small intrahepatic bile ducts. An inflammatory infiltrate is seen around the small intrahepatic bile ducts. The classic histologic finding is a florid duct lesion **(**Fig. 1), but it is only present in a minority of histologic samples [26, 28]. There is a spectrum of histologic changes. Over time, however, there is relatively slow progression from inflammatory to fibrotic change with the development of cirrhosis typically occurring within approximately 1 to 2 decades from diagnosis. However, there is wide variation in clinical presentation and course. Left untreated, there is variable progression to end-stage liver disease. While there is no cure, current treatments have been shown to delay or reduce the risk of end-stage liver disease and its complications [29].

## Need for additional treatment options

PBC carries increased risk of morbidity and mortality as well as reduced indicators of quality of life. Medical treatments for PBC are relatively new, having become available in the last 30 years. Until recently, 2016, ursodeoxycholic acid (UDCA), a synthetic bile acid, the efficacy of which will be described in full detail later in this manuscript, was the only approved medical therapy for PBC. The identification of UDCA as PBC treatment was a monumental breakthrough in medical science, but unfortunately approximately one-third of PBC patients lack an adequate biochemical response defined as a reduction in the surrogate biomarker ALP to less than 40% of baseline or less than 1.67–2 times the upper limit of normal [16]. Obeticholic acid (OCA) is the newest approved treatment, which will also be discussed later, but lacks long-term follow up on safety and efficacy. Liver transplantation has been used for the treatment of PBC-related cirrhosis, disease refractory to control by UDCA or OCA and when symptomatic treatments fail to control pruritus, but carries the real risk of transplant-related morbidity and mortality and is thus reserved only for those patients who have progressed to cirrhosis with significant liver dysfunction to warrant the surgical risk and long-term risks inherent to organ transplantation or for those patients who have developed liver-related malignancy attributed to cirrhosis or with refractory pruritus. Interestingly, even liver transplant does not appear to “cure” PBC. A small study showed that as many as 75% of patients may develop recurrent disease after transplant, though the severity of the recurrence is usually of little clinical significance [30].

## Pitfalls in developing clinical trials for PBC

There is a clear need for the development of new agents to treat PBC, but progress has been overall slow. One of the likely explanations for the difficulty in developing new and effective agents is the lack of a clear understanding of what drives the pathology of PBC. PBC is thought to progress through three phases [31]. The first is an autoimmune phase with resultant immune-mediated inflammation and damage to bile ducts. This chronic persistent damage leads to cholestasis. Bile acids are toxic to the hepatobiliary cells over time. This further drives hepatocellular inflammation. Hepatocellular and cholangio-cellular death leads to ductopenia and irreversible biliary fibrosis and cirrhosis. The difficulty in finding treatments is that many potential targets have been identified, but bile acid metabolism is complex and necessary for normal hepatic function. Complete blockage of function is not possible, as normal bile acid metabolism and homeostasis are necessary for life.

Furthermore, from a clinical standpoint, PBC represents a heterogeneous disease. While predominantly affecting middle-aged Caucasian females who possess a positive AMA, it can present in other populations and with differences in clinical severity. It is debatable whether AMA-negative and AMA-positive biliary cholangitis even represent the same disease entity, though there appears to be a genetic association. Younger age of onset and male gender as well as African and Hispanic descent portend a poorer prognosis and it is not clear why [32–35]. Biomarkers of disease severity are otherwise lacking, or in their infancy of development, though autotaxins and the microbiome are being studied as well as other potential markers [36, 37].

Additionally, the efficacy of investigational agents was limited by lack of clearly defined treatment end points. Furthermore, the development of fibrosis and the associated comorbidities of advanced liver disease such as cirrhosis-related portal hypertensive complications of portosystemic encephalopathy, development and progression of esophageal varices, and variceal bleeding, ascites formation, and hepatocellular carcinoma risk take years if not decades to occur [38, 39]. Studies require years of careful data collection and large numbers of patients, which is lacking given the rarity of PBC, to determine treatment efficacies. Consensus guidelines have been developed to help reduce the heterogeneity of therapeutic trial outcomes and to allow for standardized assessment of treatment efficacy.

In 2010, the AASLD published guidelines to set a standard for PBC trials [39]. It was realized that traditionally developed hard end points such as death or delay in the progression to liver transplant may not be the most reasonable way to evaluate drug efficacy in this condition which lacks many treatment options and takes years to decades to develop complications or lead to death or transplant. Biochemical parameters such as ALP and bilirubin, while not providing 100% correlation with histologic progression, have been considered to be reasonable indicators of clinical disease progression in PBC patients [40]. Given this recognition that biochemical end points do have the ability to predict long-term outcomes at least in the setting of UDCA response, there is the ability to design comparative clinical trials using surrogate biomarkers such as ALP and bilirubin. To date, there are several such criteria including: the Barcelona, Mayo I and Mayo II, Paris I and II, Rotterdam and Toronto criteria for response to UDCA (Table 1) [40–48]. Each has variations in the acceptable biochemical response, with all but the Rotterdam using a reduction or normalization in ALP as an essential criterion. Additionally, the development of functional scores that predict outcomes such as the GLOBE score and UK-PBC score are now also available. There are several PBC histologic scoring systems. However, given the patchy nature of the disease and the invasiveness of liver biopsy, histology is felt to be an unfavorable means to assess treatment response for any emerging therapy.

**Table 1:** Ursodeoxycholic acid response scoring criteria (alphabetical arrangement)

Scoring system	Time (months)		Alkaline phosphatase	Other biochemical markers		
				Total bilirubin	Transaminases	Albumin
Barcelona	12	Decrease 40% or normalization		–	–	–
Globe	12	2 ULN		–	–	–
Paris I	12	3 ULN		> 1 mg/dL	AST 2 ULN	–
Paris II	12	1.5 ULN		> 1 mg/dL	AST 1.5 ULN	–
Rochester I	6	2 ULN		–	–	–
Rochester II	12	2 ULN		> 1 mg/dL	–	–
Rotterdam	12	–		Normalization	–	Normalization
Toronto	24	1.67 ULN		–	–	–

The various proposed criteria for scoring a response to UDCA-treated patients are above presented. All but Rotterdam incorporates improvement in alkaline phosphatase typically as a function of upper limit of normal (ULN). Albumin, aspartate aminotransferase (AST), and albumin may also be used. The UK-PBC score also predicts outcomes in a mathematical formula derived from baseline platelet count, albumin, and 1 year total bilirubin, transaminases, and alkaline phosphatase

Currently, a search for primary biliary cholangitis or primary biliary cirrhosis in the National Clinical Trials Registry reveals slightly more than 100 clinical trials relating to the disease. Many have been completed or are not actively recruiting. Less than 20 appear actively assessing novel therapies.

## Aim of this article

The aim of this current article is to highlight the fact that PBC has few treatment options to date. Currently available treatment options while effective in some patients may lack efficacy or lead to intolerable side effects in up to one-third of patients. The main goal is to review the currently available treatment options, review those that have been shown to be less effective, and highlight the newest possible agents which may be used to treat the disease.

## The current state of PBC treatment

As it currently stands, there are three potential treatments for PBC. Ursodeoxycholic acid is the mainstay of treatment and typically considered the foundation of any PBC treatment. Failure of UDCA therapy leads to add-on therapy such as the addition of OCA which is also approved for use as monotherapy in UDCA-intolerant patients. The last currently available treatment is liver transplantation which is reserved for patients who have developed end-stage liver disease.

## Ursodeoxycholic acid (UDCA)

UDCA is a synthetic bile acid which exerts its beneficial effects through ill-defined mechanisms. It is believed to have impact on the immune system with anti-inflammatory properties, promote bile excretion, and reduce the severity of cell injury [49]. There are many formulations and dosages of UDCA available on the market without clear benefit of one over another [50, 51]. Given the possibility of gastrointestinal side effects, expert opinion recommends slow up-titration and preferably divided dosing of UDCA, though once daily dosing could be considered if compliance may otherwise be an issue. The therapeutic dose of UDCA is narrow and evidence suggests that 13–15 mg/kg/day in divided doses is beneficial. Lower doses of UDCA 5–7 mg/kg per day were found to be inferior to the 13–15 mg/kg/day dosing [52]. Higher doses of UDCA 23–25 mg/kg/day have not been shown to offer any clinical benefit [52]. While UDCA is generally thought of as a benign medication, still higher doses of UDCA 28–30 mg/kg/day have been shown to be harmful in primary sclerosing cholangitis (PSC) with an increase in hepatic decompensation [53]. An actively recruiting phase IV trial (NCT03345589) aims to assess the efficacy of an intermediate dose of UDCA 18–22 mg/kg/day compared with standard dose over 6 months of therapy with a trial end point of biochemical remission or response based on surrogate markers of liver biochemistries including ALP.

UDCA treatment is recommended in all PBC patients with abnormal biochemistries regardless of the presence of advanced liver disease, as it appears most effective in earlier stages of disease, though patients with all stages of liver disease may benefit to some degree [17, 54]. UDCA is unique as it is the one treatment for PBC with a long enough history to show an improvement in hard clinical end points such as need for liver transplant or death [55–61]. Strikingly, life expectancy of UDCA-treated patients early in the disease course may portend a life-expectancy close to the unaffected population.

While effective, up to 34% of UDCA-treated PBC patients fail to respond to treatment as defined as lack of normalization or reduction in ALP by greater than or equal to 40% at 1 year of treatment [62]. There are no clear biochemical markers to date which reliably predict response to UDCA. As such, the need for additional treatment options was recognized.

When thinking about the next steps in therapy, the emerging treatments for PBC will likely be drug regimen combinations. This is suspected because UDCA has the largest data to support its efficacy and it will be difficult to design ethical clinical trials without its use, because while only 30% of patients may fully respond to UDCA, even incomplete response has been shown to improve transplant-free survival [63]. Thus for the foreseeable future, UDCA will likely remain the backbone of any PBC regimen, until such an effective treatment is found that it will allow for trials of UDCA withdrawal to be developed.

## Obeticholic acid (OCA)

OCA became the second approved treatment for PBC in 2016. Given the urgent need for additional treatments, it was given a fast-track approval through a pathway for orphan drugs. It is approved for use in combination with UDCA, in those UDCA-treated individuals failing to achieve biochemical response after 1 year of appropriately dosed therapy. It may also be used as monotherapy for those rare patients who develop intolerable side effects to UDCA.

OCA is a potent farnesoid X receptor (FXR) agonist. FXR is responsible for modulating bile acid synthesis. FXR activation of CYP7A1 causes inhibition of bile acid synthesis [64]. In addition to inhibition of bile acid formation, OCA may also have a choleretic effect as shown by Kjaergaard et al. in vivo with ^11^C-Csar PET (AASLD 2019 #1269).

A series of clinical trials have established OCA’s efficacy as an adjunctive treatment for UDCA-refractory or intolerant PBC. The initial study of OCA was performed in 59 patients where it was used as a monotherapy in patients who had not been exposed to UDCA in the preceding 6 months prior to study enrollment. Randomized patients underwent 12 weeks of treatment with placebo, 10 mg, or 50 mg of OCA. Marked significant reductions in ALP were noted in the 10 mg and 50 mg group compared with placebo. There was no difference in biochemical outcome between the doses. However, it should be noted that approximately 40% of patients in the 50 mg OCA group discontinued therapy due to pruritus which is now a well-established side effect. A subsequent phase II study of 165 patients which did allow for the continued use of UDCA assessed outcomes of 10 mg, 25 mg or 50 mg dosages over a 3 month period [65]. Approximately 70% of patients had a treatment response, with up to a 25% decrease in ALP seen in the treatment groups, whereas less than 5% were seen in the placebo group. Pruritus remained a source of significant dropout. The POISE trial was a phase III study which established the current dosing guidelines for OCA. Patients were randomized to placebo, OCA 5 mg/day titrated to 10 mg after 6 months if lacking clinical benefit, or OCA 10 mg [66]. 210 patients were included in the trial and treated for a year. Nearly 50% of participants achieved the primary end point of reduction in ALP to less than 1.67 times ULN, reduction in ALP by greater than 15% and normal bilirubin level. Pruritus again remained the most common side effect. Patients had the option to continue in an open label extension.

Results from the open label extension of POISE trial have recently been reported. A remarkable 98% of patients were enrolled in the open label extension. In the recently published 3 year interim analysis, data from 193 patients were available [67]. At 48 months, there was sustained improvement in ALP and bilirubin compared with baseline. Pruritus continued to afflict 77% of patients, and 7 patients (4%) ultimately withdrew from the study due to pruritus. There was one death not attributable to OCA. While 8% of patients had serious adverse events, none were considered to be directly attributable to OCA. The results of 116 patients, of whom 52 had received OCA throughout the 5 years of study duration, were recently presented (AASLD 2019 #LO6). Over half of the patients continued to meet the primary end point of ALP reduction by 15% or more, with the total ALP below 1.67xULN and normal bilirubin. Mean liver stiffness scores as measured by transient elastography also remained stable. Of two deaths, neither a septic episode nor jaundice due to alcohol-related hepatitis was attributed to OCA use. Overall, 4% (8 patients) dropped out due to pruritus, with the vast majority having mild pruritus.

The safety and efficacy of OCA continue to be assessed in two phase IV clinical trials. NCT02308111, also known as COBALT, is looking to assess hard primary end points including death, transplant, and hepatic decompensation. NCT03633227 is assessing the pharmacokinetics and safety of OCA titrated to a maximum dose of 10 mg twice weekly in patients with Child B and C confirmed cirrhosis as measured by liver biopsy, transient elastography with a score greater than or equal to 16.9 kPa, or clinically apparent portal hypertension or cirrhosis based on clinical and biochemical parameters. It should be noted that there is growing evidence OCA may cause hepatic decompensation or acute liver failure requiring transplantation possibly in a dose-dependent fashion [68].

## Targets of emerging treatments and possible therapeutic agents

When approaching therapeutic targets, it is useful to review the phases of PBC. Again, there is autoimmune-mediated inflammation which leads to cholestasis, ductopenia and biliary fibrosis. Thus, these three phases represent the focus of treatment targets (Table 2).

**Table 2:** Selected emerging treatments

Immunomodulatory	Bile acid regulators	Anti-inflammatory/oxidative stress reduction
**Budesonide** Methotrexate Azathioprine Mycophenolate mofetil Targets of CXCL Targets of CTLA4 Interleukin modulation (ustekinumab) B-cell depletion (rituximab) Stem cell transplantation	Farnesoid X Receptor Agonism (FXR): **OCA**, Cilofexor, Tropifexor, EDP-305 PPAR agonists: **Fibrates**, Elafibranor, **Seladelpar**^a^ Targets of TGR-5 Targets of S1PR2 Apical sodium-dependent bile acid transporters (maralixibat) Ileal bile acid transporters	**UDCA** **SAMe** Colchicine Setanaxib Anti-fibrotic agents
		**Other agents/targets** Copper chelation Microbiome modulation

Several agents are presented with their intended target in the pathogenesis of PBC including immune system modulation, bile acid regulation and reduction of inflammation or oxidative stress. The table represents some selected treatments discussed in the text arranged by target. Agents in bold are either approved for use or have at least modest evidence to support use. Many agents and targets are still in developmental stages. Immunomodulation has largely been shown to be ineffective, though budesonide is helpful in overlap syndromes. The largest pool of agents targets bile acid regulation. Fibrates should be considered for use given growing evidence

^a^Seladelpar appears promising, but trials are currently on hold given concerns over inducing liver disease

## Immune system modulation

While the first mechanism of liver injury in PBC is immune-mediated inflammation of hepatobiliary cells, immunosuppressive treatments have largely been shown to be ineffective in isolation or as adjunctive therapies in PBC. Immunosuppressive treatments often lack specific targets and thus may have unfavorable systemic side effects such as osteoporosis [69]. However, Leuschner et al. showed a significant improvement in liver histology and ALP in their randomized trial of 39 patients, of whom 20 were randomized to receive budesonide at a dose of 3 mg three times daily over a 2 year period [70]. Rautiainen in 2005 published the results of a 3 year randomized, but open label study of patients receiving combination budesonide at a dose of 6 mg/day in combination with UDCA [71]. Liver fibrosis regressed in 25% of treated patients. The open label study which confirmed worsening of osteoporosis in treated patients showed little improvement in 22 biochemical non-responders to UDCA as evidenced by persistent elevations in ALP > 2 ULN [69]. Hirschfeld et al. presented their interim data (NCT00746486) on 62 PBC UDCA non-responders with confirmed histologic inflammation. 40 patients received budesonide administered in a 3 mg dose three times daily. There was no improvement in liver histology after a mean of 25 months of treatment. ALP dropped by 94 points, and ALP normalized in 35 versus 9% which was significant. The primary end point of ALP reduction < 1.67ULN, 15% overall reduction and normal bilirubin were achieved in over 40% of patients by 12 months, with significant difference from placebo over 3 years of treatment. There was no worsening of osteoporosis or other adverse events. Combination therapy with UDCA, prednisolone and azathioprine did improve albumin and ALP in patients with high baseline transaminases [72].

Other immunosuppressive agents such as methotrexate, a folic acid reductase inhibitor and mycophenolate, an inosine monophosphate dehydrogenase inhibitor, have been studied with conflicting results. A study by Coombes and colleagues, the PUMPS trial, examined the addition of weekly MTX to UDCA standard treatment versus addition of placebo [73]. In 132 patients who were exposed to MTX over a median of 7.6 years, and were continued until the trial was stopped as futility was documented or they underwent transplant, dosage adjustment or cessation due to MTX toxicity or development of cancer, no differences were seen in hard end points such as transplantation or decompensation. Lindor et al. and Gonzalez-Koch et al. also failed to show significant benefits of add-on MTX [74]. Kaplan and colleagues showed in a 10 year study and then a 20 year follow-up study that colchicine and methotrexate may indeed be effective in a subset of patients in improving biochemical and histologic end points, but improved mortality has not been seen [75, 76].

However, it should be noted in the initial PUMPS trial, 30% of patients stopped therapy due to adverse events [73]. A recent abstract (AASLD 2019 #1260) presented by Krampitz and colleagues showed that in a study of sub-optimal UDCA-responders with an average of 7.4 years of follow-up, MTX did improve biochemical end points with a significant 21% drop in ALP in the treated group compared with 2% in placebo, but had no effect on clinical end points. Mycophenolate has had mixed results with some studies showing improvement in histology, but another study showing no meaningful change in biochemical surrogates, but with a significant number of patient side effects [77, 78].

E6011 (NCT03092765), an anti-human fractalkine/CX3CL1 (FKN) monoclonal antibody, was evaluated in UDCA-incomplete responders. The FKN–CX3CL1 interaction appears to be overexpressed in PBC-damaged bile duct epithelial cells. In the abstract presented by Tanaka (AASLD 2019 #1266) and colleagues, 29 Japanese UDCA non-responders with elevated ALP were treated in a double blind fashion. However, this study was discontinued due to lack of efficacy after 12 week interim analysis. Other CXCL chemokine modulators have likewise failed to show significant benefit in clinical trials.

Multiple studies have looked at modulation of various components of the immune system and otherwise failed to show significant benefit. Modulators of T-cell function and of T-cell recruitment have been studied with negative results. Limited data have shown improvement in ALP with significant improvement in pruritus with rituximab but with no improvement in fatigue [79, 80]. The risks of stem cell transplantation, which has been evaluated in several studies, has failed to show clinically significant benefit and does not appear to be worth the risks of such an invasive procedure.

## Agents that reduce oxidative stress and inflammation

S-Adenosyl methionine (SAMe) is an antioxidant which increases glutathione levels. In 24 PBC patients treated with UDCA for longer than 6 months, addition of SAMe at 1200 mg/day showed significant improvement in ALP in non-cirrhotic patients as compared to age-matched controls [81]. Withdrawal of SAMe led to insignificant increases in biochemistries. In cirrhotic patients, a significant decline in bilirubin was noted. There was significant improvement with regard to pruritus and fatigue.

Setanaxib (GKT831) a NOX1/4 inhibitor which inhibits NADPH oxidases 1 and 4, involved in the development of hepatic inflammation and fibrosis, was shown to improve fibrosis and cholestasis. In a study (AASLD 2019 #1283) of 111 patients randomized to placebo, GKT831 400 mg once daily or twice daily for 24 weeks, a significant drop in fibrosis scores of about 2 and 3 kilopascals, respectively, in the 400 mg and 400 mg twice daily treatment groups compared with placebo was observed in those with a baseline liver stiffness in excess of 9.6 kPa. Overall, there were 2 adverse events in each of the treatment groups which led to drug discontinuation. Mean decreases in ALP were also significant in both treatment groups compared with placebo.

## Regulators of bile acid homeostasis

There are several important mediators of bile acid metabolism including: farnesoid X receptor (FXR), plasma membrane-bound G protein-coupled receptor (TGR5), sphingosine-1-phosphate receptor 2 (S1PR2), and peroxisome proliferator-activated receptors (PPAR) [82-84]. FXR agonism downregulates cytochrome 7A1, which is a major regulator of bile acid homeostasis. OCA, as previously discussed, is the best known FXR agonist. FXR functions in the hepatocyte to decrease bile acid synthesis, increase secretion and conjugation. In the stellate, or scar forming cells, FXR has been shown to decrease fibrosis. TGR5 is a membrane receptor for bile acids located on macrophages. In the Kupffer cell, agonism of the TGR5 receptor results in reduced cytokine production. In cholangiocytes, there is increased cell proliferation. The receptor is not however present in the hepatocyte. Sphinogsine1PR2 is also expressed in hepatocytes, Kupffer cells and cholangiocytes. In hepatoctyes, activation results in decreased bile acid synthesis and increased fatty acid oxidation. In cholangiocytes, there is an increase in cell production. Again, seen in Kupffer cells, there is a reduction in cytokine production. While these targets are exciting and there have been hundreds to a few thousand papers on these targets published in the last two decades, overall clinical utility is limited as evidenced by the relative paucity of clinical treatment trials currently ongoing.

Other possible targets are modulators of bile acid transport. The clinical reasoning here is that modulation of the concentration and composition of the bile acid pool could have positive effects at reducing the disease burden in cholestatic liver disease. Apical sodium-dependent bile acid transporter (ASBT) and ileal bile acid transporter (IBAT) inhibitors were shown to reduce plasma cholesterol levels and increase bile acid secretion [85]. Unfortunately, this led to significant side effects including choleretic diarrhea. However, some of these agents have subsequently been proven useful in chronic idiopathic constipation. Maralixibat was shown to reduce bile acid levels and improve pruritus in greater than 50% of patients; however, this did not differ from placebo [86].

## Targets of FXR

In addition to OCA, which is currently used in clinical practice, there are two other FXR agonists, cilofexor and tropifexor, which show potential promise. The results of NCT02516605 evaluating differing doses of tropifexor compared with placebo and with the notable exclusion that patients may not be on OCA have been submitted, and the results are anticipated. Additionally, EDP-305 has shown benefit in reducing liver injury and fibrosis in mouse models (AASLD 2019 #2202), and an active yet currently not recruiting phase II trial NCT03394924 seeks to assess its safety and efficacy in PBC.

## PPAR agonists

PPARs affect bile acid homeostasis, including the regulation of bile acid synthesis and detoxification. There are three isoforms, all of which have been used as therapeutic targets. Fibrates, including fenofibrate and bezafibrate, which are among some of the most promising emerging therapies for PBC are PPAR agonists. elafibranor and seladelpar also target the PPAR pathway.

## Fibrates

A pilot study of 20 patients with PBC was conducted in 2011 [87]. Patients received fenofibrate 160 mg daily in addition to standard dose UDCA for 48 weeks. ALP levels significantly dropped from a baseline mean of 351U/L to 177U/L. Cessation of treatment resulted in rebound ALP elevations. In this study, heartburn was the most common side effect. Furthermore, Ghonem and colleagues (AASLD 2019 #1263) showed that in 30 PBC patients, the addition of 160 mg/day of fenofibrate to a standard dose UDCA regimen significantly reduced ALP levels to normal ranges and improved ALT levels. Bilirubin was relatively unchanged. There was also significant alteration in the bile acid pool with reduction in pro-inflammatory cytokines.

In 2016, a large retrospective study showed improvement in hard end points of decompensation-free and transplant-free survival in addition to improved surrogate end points [62]. Of 120 patients, 46 were exposed to fenofibrate. Using the Toronto criteria for response, 41% of the fenofibrate group versus 7% in the UDCA group met criteria for a clinical response. Regardless of clinical response, exposure to fenofibrate was associated with improved transplant-free and overall survival. When comparing non-cirrhotic groups, bilirubin trended toward significantly higher in the UDCA alone group, and remained lowest in the combo group. Notably, withdrawal due to side effects was quite high, in excess of 20%, and cirrhotic patients developed more rapid increase in serum bilirubin suggesting a propensity of fenofibrate to precipitate hepatic decompensation in patients with advanced fibrosis.

In 2018, the BEZURSO study (NCT01654731) conducted by Corpechot and colleagues reported outcomes of their phase III trial of 100 UDCA-inadequate responders based on Paris II, 50 of whom were randomized to receive bezafibrate 400 mg daily plus standard dose UDCA versus standard UDCA and placebo [88]. The 24 month study showed that 31% in the bezafibrate group compared with 0% in the placebo group achieved complete biochemical response as defined by surrogate markers including bilirubin, ALP, albumin and prothrombin index time (PT). ALP normalized completely in 67% of the treated group. Pruritus also improved. Myalgias were present in 20% of treated patients, including a patient with rhabdomyolysis who was on statin therapy at the time. While liver stiffness improved in 15% of patients as measured by non-invasive means, of 28 patients with available biopsies there was no change in histology. Portal hypertension and a high alkaline phosphatase level greater than 2.53ULN at baseline were identified as independent predictors of treatment failure. Bezafibrate was associated with a 5% increase in serum creatinine, which is a known complication of PPAR-α agonists. The authors concluded that advanced cirrhosis and severe cholestasis could limit therapy with bezafibrate.

## Other PPAR agonists

Seladelpar, a PPAR delta agonist, has shown promise [89]. In a trial of 41 randomized non-UDCA responders there was a significant drop in ALP of 12 weeks in both groups on seladelpar 50 mg and seladelpar 200 mg. Pruritus was the main side effect. While all patients who completed 12 weeks of therapy had normalization of ALP, there were instances of grade 3 increases in liver injury tests to 5–20 times the upper limit of normal. Future studies are expected to evaluate seladelpar at lower doses. However, enthusiasm for this medication has very recently been tempered, as CymaBay therapeutics has announced as of November 25, 2019 a halt to all studies using seladelpar, after liver biopsies in study patients with non-alcoholic steatohepatitis and PSC revealed unexpected interface hepatitis of unclear precipitant.

Elafibranor, a PPAR-alpha/delta dual agonist, was studied in UDCA non-responders (NCT03124108). Randomized patients received 80 mg or 120 mg daily for 12 weeks. 73.3% of patients in the 80 mg group and 42.9% in the 120 mg group versus 0% in the placebo group achieved the primary end point of reduction in ALP by 40% from baseline, less than 2 × ULN, and normalization of bilirubin.

## Other agents

Several other agents have been tried and have not shown a clear benefit, or results have not been published. These include dietary and herbal supplements including mare’s milk and milk thistle. Tetrathiomolybdate, a copper chelator, appeared to reduce AST and ALT, but had no effect on ALP [38, 90]. CTLA-4 gene polymorphism in Asians, but not Caucasians may be a risk for disease development [91]. CTLA-4 inhibition has not been found to be beneficial [92]. Interleukin modulation with ustekinumab has also been found ineffective. B-cell depletion with rituximab lacks long-term efficacy and is not beneficial for the treatment of fatigue, but may improve pruritus [79, 80]. Other biologic therapies including invasive measures such as stem cell transplantation have also lacked significant enough benefit to be worth the potential side effects. Growing bodies of data suggest that liver disease may either be caused by or result in alterations in the microbiome. An ongoing study NCT03521297 will assess the efficacy of probiotics in addition to UDCA. NCT03590886 will assess other microbiome targets. Anti-fibrotic agents are being studied in several liver diseases, but insufficient data are yet available to determine their use in PBC.

## Practical treatment considerations

**Fig. 2:**
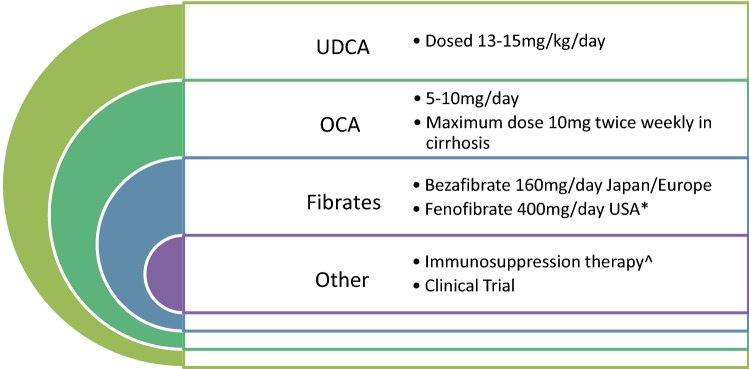
Proposed treatment algorithm for patients with primary biliary cholangitis (PBC). Ursodeoxycholic acid (UDCA) dosed at 13–15 mg/kg/day in divided doses is the standard treatment for all patients with PBC and should be initiated in all patients regardless of disease stage. In patients with incomplete response to UDCA therapy after one year, obeticholic acid (OCA) may be initiated at a dose of 5 mg/day and up-titrated to 10 mg/day after 6 months if there is incomplete response. It may also be used as monotherapy in UDCA-intolerant individuals. Extreme care should be taken when initiating this therapy in cirrhotic patients. The maximum dosage is limited to no more than 10 mg twice weekly in cirrhosis. Bezafibrate is an acceptable adjunctive therapy in Europe. It is not available in the USA. Fenofibrate (*) may be considered in an off-label use. Fibrates should not be combined with statins or used in advanced liver disease. Renal function should be monitored. Immunosuppression (^) specifically budesonide may be considered in patients with overlap PBC and autoimmune hepatitis. Other agents such as methotrexate, mycophenolate and calcineurin inhibitors have less robust evidence to support use. For patients intolerant or failing the above regimens, clinical trials may be considered

The treatment landscape for PBC has been relatively stable for the last 30 years. However, within the last 3–5 years research appears to be advancing. The mainstay of treatment is UDCA dosed at 13–15 mg/kg/day preferably in divided doses (Fig. 2). For those patients failing to achieve adequate biochemical response based on any one of several available criteria which include reductions in ALP and normalization of bilirubin in most cases, other agents should be added. In patients intolerant to or failing to adequately respond to UDCA, OCA should be considered. The initial starting dose is 5 mg/day and assessment should be made after 6 months to determine whether a dose increase is necessary. A very important caveat is that OCA needs to be markedly dose reduced in the setting of cirrhosis due to concerns over safety. Instead of daily dosing, the dose should be 5 mg once weekly, with careful up-titration to no more than 10 mg twice weekly. Pruritus remains a common side effect. Outside an overlap condition, it is not currently clear that immunosuppressive agents have a defined role in pure PBC and are not currently recommended.

While not approved in the USA, fibrates appear to be a very promising option, with both fenofibrate 160 mg/day and bezafibrate 400 mg/day showing beneficial effects on surrogate end points and symptoms including pruritus. Emerging data suggest that combination therapy with triple therapy with UDCA, OCA and fibrates may be even more beneficial in improving surrogate end points (AASLD 2019 #LB06). However, it should be noted that current guidelines recommend against the combination use of OCA and fibrates in advanced cirrhosis given the real risk of precipitating further decompensation [17]. Additionally, patients on fibrates need careful assessment of renal function, and for rhabdomyolysis. These agents should not be combined with statins. Clinical trials remain an option for patients failing or with intolerance to the aforementioned therapies.

Treatment of symptoms and metabolic complications in patients with PBC is also very important. Given the autoimmune nature of the condition, assessment should be made for other autoimmune conditions such as monitoring of thyroid function, and for sicca symptoms including dry mouth.

**Fig. 3:**
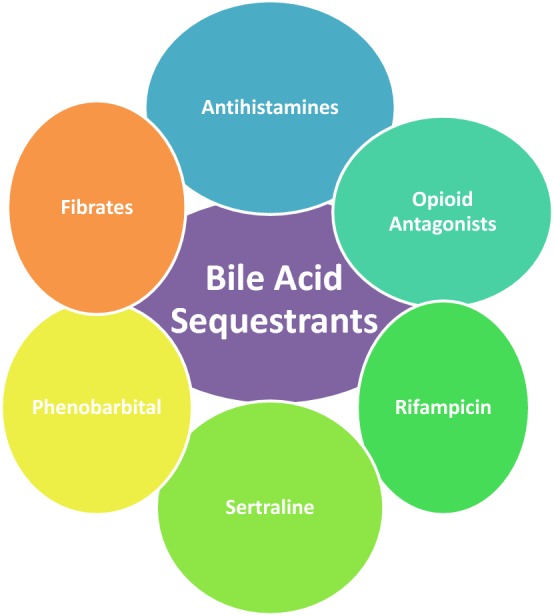
Pruritus management in PBC. Pruritus is one of the most debilitating impairments to quality of life for PBC patients. The backbone of treatment is bile acid sequestration. In patients failing to respond to therapy, other agents may be added to the regimen including antihistamines, which most likely exert positive effect through sedation, rifampicin, though care must be taken to watch for hyperbilirubinemia and hepatotoxicity, the opioid antagonist naltrexone, the selective serotonin reuptake inhibitor sertraline dosed 75–100 mg/day, which has shown effect independent of its antidepressive properties, and fibrates which appear to affect disease course and treat pruritus. Phenobarbital has been used but caution is advised given potential risk for toxicity

Pruritus and fatigue are the most common and severe complications of PBC. So far, no specific pharmacotherapy has proven beneficial in treating fatigue in PBC. Likewise, liver transplant has not been shown to improve fatigue either. Currently, a stepwise approach is recommended for pruritus (Fig. 3). Anion-exchange resins are considered the first-line treatment. Caution in proper timing of the dosage is advised, given that in addition to binding bile acids, they may also bind medications limiting their absorption. A stepwise approach thereafter with rifampicin, opiate antagonists or the selective serotonin reuptake inhibitor sertraline may also be used. Antihistamines are one of the most commonly prescribed medications and may be beneficial to help patients sleep given that sedation is a side effect. The recently reported results of the FITCH trial, which have shown bezafibrate to cause a greater than 50% reduction in pruritus symptoms in about 50% of patients, may ultimately result in fibrates being added to this algorithm (AASLD 2019 #13).

## Conclusion

The treatment landscape for PBC is changing as newer treatment targets are investigated. Currently, UDCA remains the mainstay of treatment with adjunctive therapies added when incomplete response is achieved. Despite three phases of PBC, immune, cholestasis-induced injury and fibrosis, the cholestasis-induced injury seems to be the most promising physiologic target given the lack of data to support immune-system modulation and lack of studies evaluating anti-fibrotic therapies.
